# An Unusual Case of Ehlers-Danlos Syndrome Presenting as Proptosis

**DOI:** 10.7759/cureus.41715

**Published:** 2023-07-11

**Authors:** Sanjay M Khaladkar, Suhas M, Rajshree Dhadve, Udayan Dosi

**Affiliations:** 1 Radiodiagnosis, Dr. D. Y. Patil Medical College, Hospital & Research Centre, Dr. D. Y. Patil Vidyapeeth, Pune, IND

**Keywords:** proptosis, joint hypermobility, skin hyperelasticity, caroticocavernous fistula, vascular ehlers danlos syndrome

## Abstract

Ehlers-Danlos syndrome (EDS) is a rare, heterogeneous group of genetic connective tissue disorders that affect collagen proteins. Currently, they are classified into 13 subtypes, many of which share general characteristics such as thin, hyperextensible skin and joint hypermobility. Vascular Ehlers-Danlos syndrome (vEDS) is characterized by tissue fragility, which predisposes individuals to premature arterial, uterine, or intestinal rupture. In this case, a young female presented with proptosis, skin hyperelasticity, and multiple joint dislocations. On computed tomography angiography (CTA), a direct caroticocavernous fistula, along with multiple segments of narrowing and ectasia in the internal carotid arteries and vertebral arteries, were detected, leading to a diagnosis of vEDS. This case report highlights the importance of clinical evaluation and the role of imaging in detecting this rare condition.

## Introduction

Ehlers-Danlos syndrome (EDS) is a rare, heterogeneous group of inherited connective tissue disorders with a distinct genetic basis that affects specific collagen proteins. Currently, EDS is classified into 13 types. Many types share general characteristics like joint hypermobility, skin hyperextensibility, and tissue fragility. Each type has a set of clinical criteria in the form of major and minor criteria to guide the diagnosis [1]. 

Vascular Ehlers-Danlos syndrome (vEDS) occurs due to mutations in the COL3A1 and/or COL1A1 genes. This impacts the synthesis and structure of the pro a1 (III) chain of collagen type III, leading to weakness of the vessel wall [1-3]. Clinically, the patient has thin, translucent skin, easy bruising, acrogeria, and characteristic facial features: thin lips, a narrow nose, and prominent eyes. Classical features are intestinal or uterine fragility, but most commonly, arterial fragility is seen, presenting with spontaneous dissections, aneurysms, and arterial ruptures [1]. The diagnosis is confirmed by analyzing type III procollagen produced by cultured fibroblasts and identifying the COL3A1 gene mutation [1,4].

## Case presentation

A young 30-year-old female presented with a long-standing history of left proptosis, redness in both eyes, and right lateral rectus palsy for two years. She also had a congenital right clubfoot. On examination, she had skin hyperelasticity and thinning and joint hypermobility. On radiography, she was found to have arachnodactyly and dislocations at the left elbow and right metatarsophalangeal joints. There was no family history of a similar condition. 

**Figure 1:**
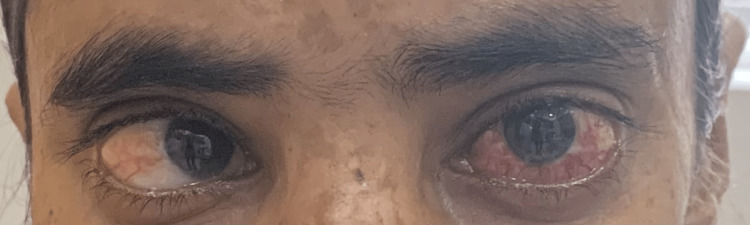
Clinical image The clinical picture shows chemosis and proptosis of the left eye and adduction of the right eyeball

Figure 1 shows a clinical picture depicting chemosis and proptosis of the left eye, as well as adduction of the right eyeball.

**Figure 2:**
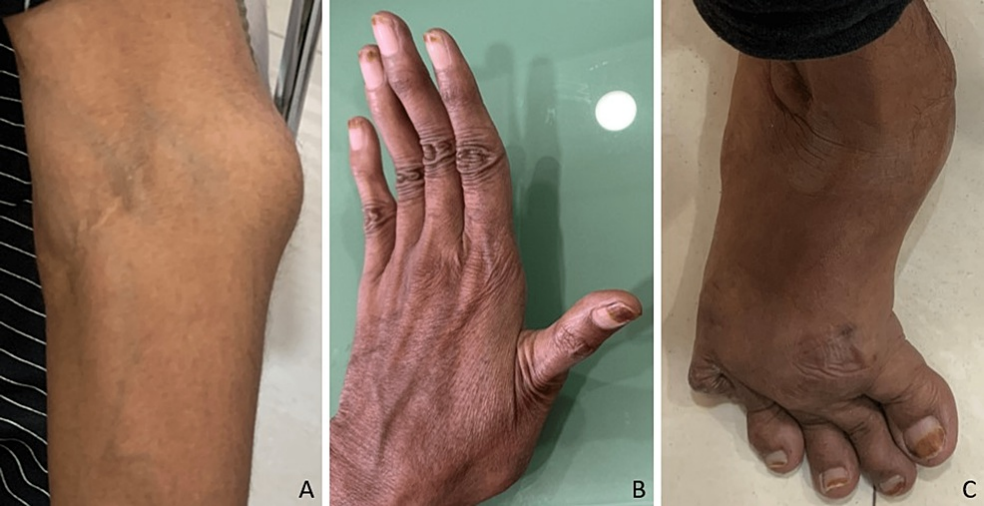
Clinical images The clinical picture shows subluxation of the left elbow (A), skin hyperelasticity and joint hypermobility (B), and a right clubfoot with deformity caused by dislocations at the right metatarsophalangeal joints (C)

Clinical images in Figure 2 illustrate the following conditions: subluxation of the left elbow (A), skin hyperelasticity and joint hypermobility (B), and a right clubfoot with deformity caused by dislocations at the right metatarsophalangeal joints (C).

**Figure 3:**
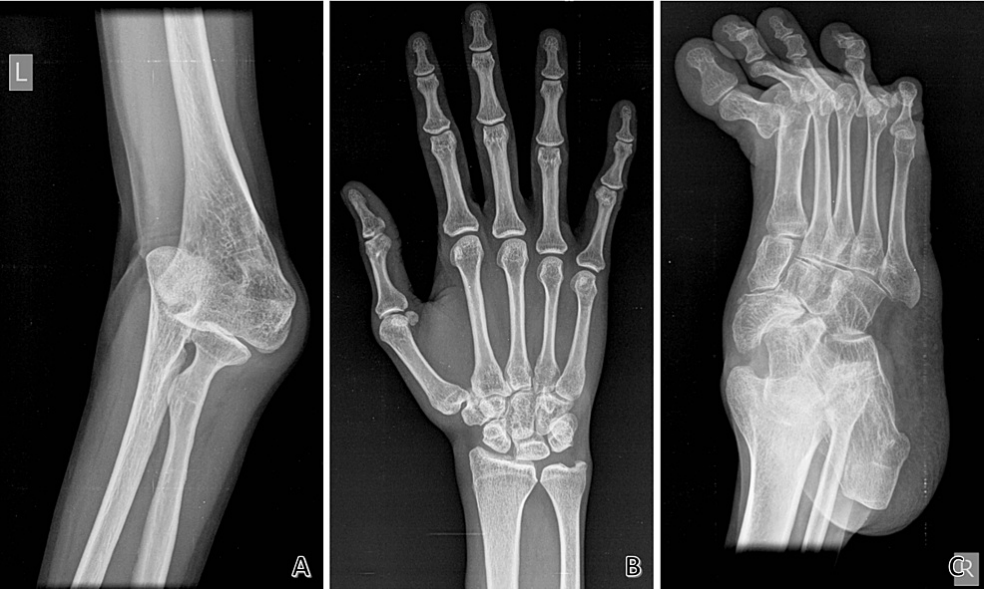
Radiographs The radiographs show subluxation of the left elbow (A), arachnodactyly (B), and dislocations at the right metatarsophalangeal joints (C)

Figure 3 shows radiographs depicting joint dislocations (A, C) and arachnodactyly (B).

Her magnetic resonance imaging (MRI) and magnetic resonance angiography (MRA) of the brain revealed a direct-type communicating fistula between the left cavernous sinus and the cavernous part of the left internal carotid artery. Venous drainage was mainly into the left superior ophthalmic vein and the internal jugular vein through the inferior petrosal sinus. Multiple fusiform aneurysms were seen along the cervical part of the left internal carotid artery and the V3 segments of the left vertebral artery, giving the appearance of a string of beads. No flow-related enhancement of the right internal carotid artery was noted after its origin from the right common carotid artery, suggesting complete occlusion. Cystic encephalomalacia with surrounding gliosis was noted in the posteroinferior part of the right cerebellar hemisphere, suggesting an old infarct.

**Figure 4:**
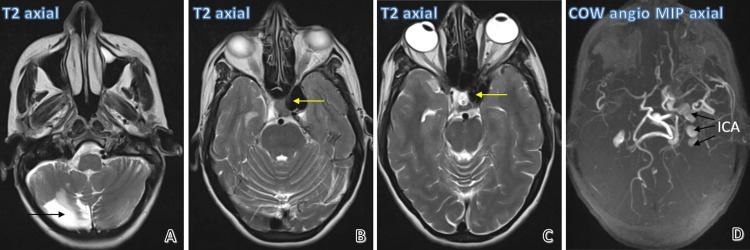
MRI and MRA findings MRI images show cystic encephalomalacia in the right cerebellar hemisphere (black arrow) (A), large flow voids (yellow arrows) in the cavernous sinus (B) and ICA (C), and the MIP image of the circle of Willis (COW) angiography shows fusiform aneurysms in the ICA (black arrows) (D).

Figure 4 shows MRI images depicting cystic encephalomalacia in the right cerebellar hemisphere on a T2-weighted image (A), flow voids due to direct caroticocavernous fistula on T2-weighted images (B, C), and a maximum intensity projection (MIP) image of circle of Willis (COW) angiography showing fusiform aneurysms in the left ICA (D).

A computed tomography neck and brain angiography (CTA) was performed to study vascular complications. The left vertebral artery originates directly from the aorta. It showed marked circumferential narrowing in its pre-foraminal segment (V1 segment) till its entry in the foramen transversarium at the C5-C6 level. Its foraminal segment (V2 segment) and atlantic segment (V3 segment) showed intermittent dilatation and narrowing. Its intracranial segment (V4 segment) appeared normal. The right vertebral artery in its V1 segment showed marked intermittent dilatation and narrowing; the V2 and V4 segments appeared normal in caliber, and the V3 segment showed mild dilatation. The basilar artery and bilateral posterior cerebral arteries appeared normal in caliber and opacification. The left common carotid artery (CCA) and the external carotid artery (ECA) appeared normal. The left internal carotid artery (ICA) in its cervical portion showed marked intermittent dilatation (ectasia) and short-segment narrowing. The horizontal portion of the left ICA appeared normal. The cavernous portion of the left ICA showed aneurysmal dilatation with a linear filling defect due to dissection. There was communication between the cavernous segment of the left internal carotid artery and the left cavernous sinus, suggesting a caroticocavernous fistula (type A). The left cavernous sinus showed outward bulging and contrast enhancement on angiogram images. There was enhancement and dilatation of the left superior ophthalmic vein and the left superficial middle cerebral vein. The left ophthalmic artery appeared prominent. The right CCA and ECA were normal. The right ICA shows mild fusiform dilatation over a length of approx. 1.7 cm, beyond which it shows marked narrowing with near complete occlusion. Horizontal and cavernous portions of the right ICA were not seen and were non-opacified. The supraclinoid portions of the right ICA, right ACA, and MCA were opacified and appeared normal (via the circle of Willis). An old infarct with gliosis in the right cerebellar hemisphere, left proptosis, and persistent adduction of the right eyeball was seen on the CT brain.

**Figure 5:**
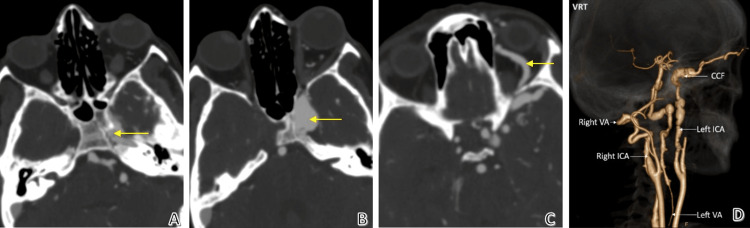
CT angiography findings Post-contrast CT images show a linear filling defect in the left cavernous ICA (yellow arrow) due to dissection (A), outward bulging of the left cavernous sinus (yellow arrow) due to caroticocavernous fistula (B), a prominent left superior ophthalmic vein (yellow arrow) (C), and a VRT image depicting narrowing and ectasia at various levels in both the carotid and vertebral arteries (D).

Post-contrast CTA images in Figure 5 depict dissection of the left cavernous ICA (A), outward bulging of the left cavernous sinus due to caroticocavernous fistula (CCF) (B), a prominent left superior ophthalmic vein (C), and a volume rendering technique (VRT) image depicting narrowing and ectasia at various levels in both the carotid and vertebral arteries (D).

The patient was advised to undergo a skin biopsy and genetic testing, but the request was denied. The diagnosis of vascular Ehlers-Danlos syndrome was made based on the presence of a non-traumatic direct left caroticocavernous fistula (type A), along with multiple segments of narrowing and ectasia observed in both the ICA and vertebral arteries. Additionally, the patient exhibited skin hyperelasticity and joint hypermobility. Treatment options, including interventional procedures, were explained to the patient, but she has currently declined them.

## Discussion

Direct carotid-cavernous fistula (CCF) (Type A) is a high-flow lesion that occurs when the cavernous part of the internal carotid artery (ICA) ruptures directly into the cavernous sinus (CS). The most common cause of this condition is trauma, accounting for 75% of cases, and often associated with central skull base fractures. Spontaneous rupture accounts for 10% of cases, while pre-existing cavernous ICA aneurysms contribute to 2%-9% of cases. Other causes include connective tissue disorders (such as vascular Ehlers-Danlos syndrome) and fibromuscular dysplasia [5].

Ehlers-Danlos syndrome (EDS) is a heterogeneous group of connective tissue disorders that cause fragility in connective tissues and can lead to life-threatening complications, particularly in young adults. The new classification (International EDS Consortium classification system 2017) recognizes 13 subtypes: classical EDS (autosomal dominant; AD), classical-like EDS (autosomal recessive; AR), cardiac-valvular (AR), vascular EDS (AD), hypermobile EDS (AD), arthrochalasia EDS (AD), dermatosparaxis EDS (AR), kyphoscoliotic EDS (AR), brittle cornea syndrome (AR), spondylodysplastic EDS (AR), musculocontractural EDS (AR), myopathic EDS (AD or AR), periodontal EDS (AD) [1]. 

The major criteria for vascular Ehlers-Danlos syndrome (vEDS) include the following: an arterial rupture in young people, sigmoid colon rupture without known diverticula or other bowel pathologies, uterine rupture in the third trimester in patients without a previous cesarean section (C-section), and/or severe peripartum tears in the perineum, non-traumatic carotid-cavernous fistula formation, family history of vEDS with documented COL3A1 gene mutation [1]. 

The minor criteria for vascular Ehlers-Danlos syndrome (vEDS) include the following: thin, translucent skin, characteristic facial features, early-onset varicose veins (occurring before the age of 30, and being nulliparous if female), hypermobility of small joints, non-traumatic bruising at unusual sites like the back and cheeks, acrogeria, talipes equinovarus (clubfoot), congenital hip dislocation, gingival recession and gingival fragility, keratoconus, tendon, and muscle rupture, and spontaneous pneumothorax [1]. 

The minimal criteria suggesting vascular Ehlers-Danlos syndrome (vEDS) include a family history of vEDS, arterial dissection or rupture in patients younger than 40, spontaneous pneumothorax, unexplained sigmoid colon perforation, and other 'minor' clinical features. These criteria should prompt diagnostic testing to confirm the diagnosis of vEDS in the patient [1].

The clinical diagnosis of vascular Ehlers-Danlos syndrome (vEDS) can be challenging, even for experienced clinicians. The diagnosis of vEDS relies on the identification of a causative variant in the COL3A1 gene for treatment and assessing the risk of recurrence [1]. Treatment options for vEDS include medical or surgical management to address arterial complications, uterine rupture during pregnancy, or bowel rupture [6].

Intracranial arteries, in contrast to extracranial arteries, have fewer elastic fibers and thinner collagen fibers in the media and adventitia layers, and they lack an external elastic lamina. As a result, they are more susceptible to rupture or dissection [7,8]. A literature review emphasizes that the development of direct spontaneous carotid-cavernous fistula (sCCF) is more common in female patients with vascular Ehlers-Danlos syndrome (vEDS) than in males [4]. In vEDS, direct CCF formation occurs due to the spontaneous rupture of the tortuous internal carotid artery (ICA) as it emerges from the petrous bone into the carotid sinus, with or without a pre-existing intracranial ICA aneurysm or as a result of spontaneous ICA dissection leading to pseudoaneurysm formation [4,9,10]. 

Interventional procedures for carotid-cavernous fistula (CCF) can include trans-arterial approaches, although they are contraindicated in cases of vascular Ehlers-Danlos syndrome (vEDS). Alternatively, a transvenous approach utilizing the inferior petrosal sinus or the superior or inferior ophthalmic vein may be employed for endovascular embolization in some cases. However, it is important to note that interventional procedures in patients with EDS can potentially increase collagenase activity, which can lead to complications occurring in rapid succession. These complications may include remote and delayed arterial rupture during or after endovascular procedures [11].

## Conclusions

Ehlers-Danlos syndrome (EDS) is a group of inherited connective tissue disorders with a distinct genetic basis that affect specific collagen proteins. Vascular EDS (vEDS) is characterized by premature arterial, uterine, or intestinal rupture. Suspicions of EDS should arise in patients presenting with thin skin, joint dislocations, and specific facial features such as thin lips, a narrow nose, and proptosis. MR brain and angiography, as well as CT angiography, are imaging techniques that can help identify specific abnormalities in patients with vascular Ehlers-Danlos syndrome (vEDS). These imaging studies may reveal narrowing and ectasia at different levels in the carotid and vertebral arteries, as well as dissection in the internal carotid artery (ICA) and the presence of a spontaneous direct carotid-cavernous fistula (CCF) in young patients. It is important to note that in the case of vEDS, trans-arterial interventional procedures for CCF are contraindicated. This is because such procedures have been observed to increase collagenase activity, which can result in complications such as arterial rupture during or after endovascular procedures.
